# Editorial: Inter-Organelle Calcium Communication in Cancer

**DOI:** 10.3389/fonc.2018.00014

**Published:** 2018-02-06

**Authors:** Cesar Cardenas, Paolo Pinton, Geert Bultynck

**Affiliations:** ^1^Anatomy and Developmental Biology Program, Institute of Biomedical Sciences, University of Chile, Santiago, Chile; ^2^Geroscience Center for Brain Health and Metabolism, Santiago, Chile; ^3^Buck Institute for Research on Aging, Novato, CA, United States; ^4^Department of Chemistry and Biochemistry, University of California, Santa Barbara, Santa Barbara, CA, United States; ^5^Department of Morphology, Surgery and Experimental Medicine, Section of Pathology, Oncology and Experimental Biology, Laboratory for Technologies of Advanced Therapies (LTTA), University of Ferrara, Ferrara, Italy; ^6^Maria Cecilia Hospital, GVM Care & Research, E.S: Health Science Foundation, Cotignola, Italy; ^7^Laboratory of Molecular and Cellular Signaling, Department Cellular and Molecular Medicine, KU Leuven, Leuven, Belgium; ^8^Leuven Kanker Instituut (LKI), KU Leuven, Leuven, Belgium

**Keywords:** mitochondria, endoplasmic reticulum, MAMs, IP3R, mitochondrial Ca2+ uniporter, BCL-2, metastasis, invasion

**Editorial on the Research Topic**

**Inter-Organelle Calcium Communication in Cancer**

## Introduction

Cancer is a leading cause of death worldwide, accounting for 8.2 million deaths (around 13% of all deaths) in 2012, with this number expected to rise to over 11 million by 2030 (1). The term “cancer” groups over 100 distinct diseases that share a series of “acquired capabilities,” which were first defined by Hanahan and Weinberg in 2000 consisting of six hallmark alterations in cell physiology that collectively dictate malignant growth: cell sufficiency in growth signals, insensitivity to growth inhibitory signals, evasion of programmed cell death, limitless replicative potential, sustained angiogenesis, and tissue invasion and metastasis (2). In the last 25 years, the description and interpretation of genomic abnormalities in cancer cells have dominated cancer research (3). However, bioinformatic analyses suggest that cancer-related driver mutations affect a dozen or more core cell signaling pathways (4), suggesting that individual targets will not be fruitful candidates for therapeutic development, and instead, intervening with cellular processes will be more effective. In this regard, it has become increasingly clear that many key oncogenic signaling pathways that support tumor cell growth and survival converge at cell metabolism (5). In fact, the reprogramming of energy metabolism, which relies on mitochondrial function, is absolutely required for malignant transformation, and for this reason, it has recently been added as a hallmark of cancer (6). This research topic focuses on the role of the endoplasmic reticulum (ER)-to-mitochondria communication in the regulation of mitochondrial function, with special emphasis on Ca^2+^ transfer, and its impact on cancer cell fate.

## Mitochondria-Associated ER Membranes in Cancer

It has been known since the 1950 that mitochondria and the ER, the main intracellular Ca^2+^ store, interact with each other in response to changes in cellular metabolism (7). The ER–mitochondrial association can be physically isolated (8) as mitochondria-associated ER membranes that establish a specific microdomain with distinct signaling functions (9). These contact sites not only allow “quasi-synaptic” transfer of Ca^2+^ from the ER to the mitochondria but also enables lipid synthesis and exchange, mitochondrial trafficking, cell death, bioenergetics, proteostasis, and autophagy, all processes often altered in oncogenesis and cancer, as has been reviewed in detail by Sassano et al. Consistent with the pivotal role of MAMs in these fundamental cellular processes, their function and thus composition ought to be fine-tuned. This is achieved by a growing list of proteins that impact cell survival and cell stress tolerance, of which, many are well-known oncogenes and oncosuppressors such as p53, AKT, PTEN, and PML (10, 11). Pedriali et al. discuss how these proteins modulate Ca^2+^ homeostasis. In addition, they present an exhaustive summary of drugs that target Ca^2+^ transport being used as anticancer therapies. Also, several Bcl-2-family members, critical regulators of cell death and often dysregulated in cancer, have been identified at the MAMs, impacting cell survival through the regulation of ER-mitochondrial Ca^2+^ dynamics, a topic further contemplated by Vervliet et al. Herrera-Cruz and Simmen go a step further and consider the role of structural proteins that enable the formation of MAMs; e.g., mitofusin-2, phosphofurin acidic cluster sorting protein 2 (PCS-2), and Nogo-B/reticulon-4B as oncogenes or oncosuppressors. As mentioned, MAMs actively participate in the regulation of proteostasis, tuning the ER stress response to cope with metabolic demand under various stress stimuli. Carreras-Sureda et al. immerses us in the unfold protein response, its relation with MAMs, and the role this plays in cancer.

## Ca^2+^ Basic Toolkit; IP3R, VDAC, and Mitochondrial Ca^2+^ Uniporter (MCU) in Cancer

Ca^2+^ is a ubiquitous signaling messenger that regulates various cellular processes ranging from muscle contraction to synaptic transmission and from cellular proliferation to cell death (12, 13). This versatility is achieved by the interplay of the Ca^2+^-signaling toolkit (including channels, pumps, exchangers, and binding/buffering proteins), generating specific spatiotemporal Ca^2+^ signals (14). Alterations in the expression and/or function of these Ca^2+^-transport/binding systems have been implicated in oncogenesis and cancer progression (15, 16). In this regard, Wolf and Guse contribute a comprehensive review focused on the generation, regulation, and function of Ca^2+^ microdomains in T lymphocytes. Ca^2+^ microdomains occur in cancer cells due to the spontaneous opening of a single or some small clusters of the inositol 1,4,5-trisphosphate (IP3) receptor (IP3R), in the ER, which proves to be necessary to maintain the bioenergetic homeostasis of these cells (17), as deliberated by Bustos et al. Ca^2+^ regulates bioenergetics and metabolism by entering the mitochondria where it modulates the activity of key enzymes of the tricarboxylic acid cycle and fatty acid oxidation (18). Ca^2+^ crosses the mitochondrial outer membrane through the voltage-dependent anion channel 1 (VDAC1), a highly Ca^2+^ permeable channel that controls metabolism and bioenergetics by modulating the access of Ca^2+^ as well as pyruvate, malate, succinate, and NADH to the inner mitochondrial membrane. Shoshan-Barmatz et al. review the role of VDAC1 in cancer with special emphasis on its role in apoptosis induced by [Ca^2+^]-increasing agents, which upregulate VDAC1 expression and subsequently results in VDAC1 oligomerization, the release of cytochrome C & Smac/Diablo, and eventually apoptosis. Once present in the mitochondrial intermembrane space, Ca^2+^ is taken up into the mitochondrial matrix through the MCU complex, whose structure, activity, and regulation has been explained by Mammucari et al. In addition, the authors highlighted the divergent roles of MCU in cancer, revealing both anti- and pro-tumorigenic roles dependent on the cancer type and cancer stage. Finally, Chen and Boehning assess the role of lipidation in the regulation of different signaling pathways that impact Ca^2+^ homeostasis, such as Ras in apoptosis and tumor progression.

## Mitochondrial Ca^2+^ Dynamics in Invasion and Metastasis

Mitochondria are highly dynamic organelles undergoing constant fusion and fission governed in part by the metabolic status of the cell (19). Cancer cells, which are characterized by alterations in their metabolism, also exhibit several changes regarding mitochondrial dynamics, morphology, and cellular positioning, as discussed in the review by Pendin et al. These alterations enable cancer cells to avoid apoptosis and to cope with the sustained energy demand, particularly during migration, invasion, and metastasis. White concisely reviews the key steps controlled by Ca^2+^ during the invasion-metastasis cascade and reveals how changes in the Ca^2+^-handling components that reside in the MAMs might facilitate invasion and metastasis by impacting bioenergetics and reactive oxygen species generation. Along these lines, Hegedüs et al. provide experimental evidence that enhanced clearance of Ca^2+^ by increased expression of the plasma membrane Ca^2+^ ATPase 4b induced by histone deacetylase inhibitors, and reduces migration in a melanoma cell line.

## Perspectives

Cancer has a multifactorial nature. Over the last decade, it has become increasingly clear that ER-to-mitochondria communication, in particular, through the flux of Ca^2+^ ions, impacts a plethora of cancer hallmarks and plays a critical role in oncogenesis. The emerging insights at the molecular level in the dependence of cancer cells on ER-mitochondrial Ca^2+^ signaling for their survival and migration will pave the path for new therapeutic strategies and tools (20), hopefully opening up new avenues for designing anticancer treatments that exploit these dependencies and vulnerabilities.

## Author Contributions

CC drafted the manuscript that was reviewed and edited by PP and GB. All authors co-edited the Research Topic.

## Conflict of Interest Statement

The authors declare that the research was conducted in the absence of any commercial or financial relationships that could be construed as a potential conflict of interest.
